# On the Lesions and Pathology of the So-called Posterior Paralysis of the Horse

**Published:** 1881-07

**Authors:** William Henry Porter, George H. Parkinson

**Affiliations:** Professor of Surgery at the Columbia Veterinary College and School of Comparative Medicine; Middletown, Connecticut


### ART. XIV.—ON THE LESIONS AND PATHOLOGY OF THE SO-CALLED POSTERIOR PARALYSIS OF THE HORSE.

POSTERIOR paralysis is a subject of great interest to the equine practitioner and horse owner, both from its great frequency and fatality. In works on veterinary practice it is apt to be associated either with "spinitis" (Williams) or azoturia, but in either case the pathology of the affection is more or less obscure.

Undoubtedly, in a few instances the exciting cause may be traced to a fractured spine, or possibly even to vascular changes, such as occlusion of the iliac arteries, from embolism or thrombosis; but these exceptional conditions are of no account in explaining the occurrence of the disease as we usually find it in practice. In the case of a fracture there may be some show of reason for calling the affair, as Williams does, a "spinitis" (a word of vicious construction, part being Latin and part Greek), which really can only be translated so as to mean an inflammation of the bony canal. But it cannot be applied to any other pathological process, and therefore is absolutely incorrect when used to indicate a meningitis or myelitis, or both. The impropriety of assuming a spontaneous origin of the disease is further shown by examining the causes that are given for the human disease.

These are either direct injury, the extension of an inflammation from contiguous parts, syphilis, venerial excesses, hemorrhage, etc., while spontaneity is rather inferred than affirmed, and then only in a small proportion of the cases. Is there, then, any special reason why one should believe that the disease is primary or idiopathic, and has no cause that we can explain, or is it not probable rather that a well-directed and intelligent search will reward us with its discovery?

*Azoturia* is another one of those convenient words which has a useful place in mystifying, but not explaining, the real condition of things. Posterior paralysis is spoken of as dependent on "nitrogenous urine," and a "hyper-nitrogenous" condition of the blood, but from physiological laws the opposite condition might often be expected when an animal, as is generally the case when paralyzed, has been at rest and fed upon a vegetable diet. The pathology of the disease, as heretofore given, is thus seen to be in a very unsatisfactory state.

Posterior paralysis is classed in various ways by different writers. Thus it is called sporadic or epidemic, according to a common division observed in infectious diseases. It is also acute or chronic. An attack is usually sudden, frequently after exposure to a sudden change in temperature. Mares are more frequently attacked than geldings, and it is apt to occur at the period of œstrum.

So far as personal experience goes, derived from a number of autopsical examinations, in cases where the existence of the disease had been established clinically by others, it leads me to the belief that serous or purulent exudation, which is the marked phenomenon where serous membranes have been inflamed, is not to be found in this disease. It is true that I have seen in some instances a marked turgescence of the vessels in the membranes of the cord, and some fluid in the subarachnoid space, and there is in some instances vascular distention of the vessels of the cord in the lumbar region. I believe, however, that these conditions indicate an active congestion or hyperaemia, with subsequent transudation of fluid, rather than an established inflammation. I must, therefore, decide from my experience that the pathological changes in those cases, except in the rare instances already alluded to, are a marked congestion of the spinal membranes lying in contact with the lumbar portion of the cord, and of the cord itself at this point. The congestion may extend along the membrane covering the roots of the spinal nerves up to the point where they penetrate the dura-mater, *where it is most intense*, and looks as if it might produce considerable pressure on the nerves. The dural layer of the arachnoid, and the pia-mater, do not appear to be congested.

In the lumbar and sacral regions, the former especially, I have found the surface of transverse sections studded at varying intervals with bright red points, and lines marked in the gray columns, both indicating vascular engorgement.

Microscopical examination of these areas revealed distended capillaries packed with blood disks, while occasionally little extravasations occurred about the vessels. It seems highly probable that, during life, an œdematous condition of the cord at this point may also accompany the congestion. The changes are most marked, as I have said, in the lumbar region; it is slight in the sacral, and absent altogether in the dorsal and cervical portions of the cord.

It is noteworthy, too, that all the cases which came under our observation exhibited extensive lesions in the genito-urinary tract. The mucous membrane of the bladder was inflamed, and often coated with a thick, ropy mucous; frequently there was an abundant loss of substance, resulting in ulcers of varying size and shape. Sometimes the inorganic salts, principally the lime carbonates, were deposited and intermixed with the ropy mucous, so as to form a pasty mass like clay. If such a case terminated in recovery, a stone in the bladder might fairly be expected, for it seems hardly possible that the bladder could rid itself unaided of so firm and large a substance.

The ureters also are congested, and sometimes inflamed, and occasionally dilated. The pelves of the kidneys frequently contain purulent fluid, the mucous membrane being congested and studded with bright red, ecchymotic spots.

The kidneys are found either hard or soft, but always very dark in color. The capsule often non-adherent, the underlying renal surface smooth but deeply congested. When the kidneys are laid open, the cut surface is dark in color, with bright red lines running in the direction of the collecting uriniferous tubules of the pyramids. The congestion is most marked in the pyramidal portion. A bloody, tenacious fluid may be seen to exude from the cut surface, which coagulates rapidly into white, flaky masses upon the addition of alcohol.

Microscopical examination shows that the most marked and constant lesion is that of an acute parenchymatous nephritis. The epithelium is swollen, cloudy, or even granular; in many places the cells are entirely stripped from the basement wall. Sometimes the interstitial tissue is slightly involved, but this is not a constant appearance. The blood-vessels are not specially distended with blood corpuscles, but numerous blood disks are to be seen scattered through the section; some pigment matter probably from the blood, is also quite abundant.

Sero-sanguinous effusions into the lumbar muscles are met with in some of the cases. In one animal there was considerable bloody effusion into the peritoneal cavity; this latter, however, was noticed in a mare at the period of oestrum, and may be regarded as an exceptional case. This hemorrhage into the peritoneal or pelvic cavity is similar to the pelvic hematocele occasionally met with in human practice. This hematocele may have been only an accidental complication.

The kidney lesion was marked in all the instances observed, though the spinal congestion was at first, if present, entirely overlooked.

The question now arises, which lesion is primary, and how can its production be explained?

To us it seems quite evident that the renal disease is secondary or complicating, and the spinal congestion and oedema the primary lesion. One reason for this view is the fact that the paraplegia almost invariably develops first, and the urinary symptoms show themselves later. Proof of this statement appears especially strong in a case reported in the Case Department by Dr. Parkinson. The paralysis of the bladder, with urinary retention, but at first no marked changes in the urine, point, it seems to me, to the spinal lesion as the primary trouble. If the renal disease was primary, it would seem reasonable to suppose that some renal symptoms would be detected prior to the paralysis. Again, if the spinal congestion was secondary, why should it be localized in the lumbar and the sacral regions, and the rest of the cord remain normal; more especially if, as is claimed, it is due to the retained products of tissue metamorphosis?

It is hard to understand how the condition known as azoturia can produce the posterior paralysis and nephritis. It may be possible that a condition of the blood exists with this disease which produces spinal congestion and nephritis. It does not seem very clear, however, how a "hypernitrogenous condition of the blood" should produce nephritis, for the amount of work to be performed by the kidneys is subject to constant variations. It is barely possible that a surcharging of the blood with effete material may irritate the spinal centres in this region, and thus produce congestion, oedema, paralysis, and nephritis; but why the lesion should be located at this given point is the puzzling question; for in human medicine, when the kidneys give way, and the products which should be excreted are retained, and the cerebral as well as the spinal centres are implicated.

The question arises in our minds if there is not a better way to explain the cause of these attacks. The sudden work required of the posterior extremities after rest produces an intense reflex irritability of the spinal centres supplying them, which is speedily followed by congestion and oedema, paralysis, and all its disastrous results. During the period of œstrum, there is probably some irritation of the spinal centres at this point, and a very little increase of this irritation may produce a decided congestion. The application of cold when under exercise seems sufficient to bring on the lesion. In all the cases active exercise of the posterior extremities appears essential to the production of the paralysis, for at rest these attacks never occur. Sudden exercise after rest, calling out the activities of the posterior cord, especially in cold weather, and during the period of œstrum, seem to be almost enough factors to explain the production of a spinal congestion and œdema.

The symptoms are not always the same during the period ot invasion, so that we have several types or sets of cases of the same disease.

One set of cases is met with in mares at the period of œstrum, especially if the animal be taken out and worked after a few days of rest. If the first day be wet or cold the greater the danger. Such an animal, you will notice, suddenly becomes weak, and staggers in the posterior extremities only; finally, there is complete loss of motion and sensation. The lumbar regions often swell up, and the extremities are sometimes cold. The pulse rises from 20 to 30 beats per minute; respiration becomes frequent and jerking; the temperature rises one or two degrees; sometimes as high as 105° or 106° F. After an hour or so, the animal strains and ejects a large quantity of dark, coffee-colored urine, which contains a large admixture of blood. It also perspires freely. The intensity of these symptoms frequently abates in 24 or 36 hours, and the animal appears to be on the road to recovery, but at the end of a day or two it dies; less often it may linger a week or more. The cause of death is exhaustion or suppression of urine, with uræmia. Sometimes recovery takes place, but the posterior extremities remain quite weak.

A second set of cases is met with both in the mare and gelding after the animal has been stabled for a few days, and then taken out and driven. To this class the name *azoturia* has been applied. The animal may be unusually spirited, and make the first few miles without showing any debility, but suddenly it appears weak in the posterior extremities, staggers, becomes restless, perspires freely, and eventually manifests a strong desire to lie down; then loss of motion and sensation is apparent, though there is often for a time spasmodic muscular contraction. The pulse rises to 60 or 80 beats per minute, and respiration is increased in frequency. When the paralysis is complete they often try to rise, but fail to get upon their feet. After straining violently, they void at intervals large quantities of dark, coffee-colored urine, containing blood. The intensity of the symptoms may subside for a time, but often the quantity of the urine gradually diminishes, and they finally die from suppression, Ultimate recovery is also rare in this form.

A third set of cases is seen after exposure to cold and wet, and therefore most frequent in the fall and spring, when cold rains are common. Similar attacks may occur in summer, when the animal has been out in the hot sun, and is suddenly caught in a thunder shower, the rain falling upon the previously heated loins. After such exposures the animal suddenly, or at the end of a few hours, becomes weak, staggers, and loses power of motion and sensation in the posterior extremities. In some of these cases there is a copious discharge of dark-colored urine, in others there is suppression from the first. The temperature, pulse, and respiration rise, accompanied by great restlessness, glassy eyes, and in some cases swelling over the loins. In these cases the bowels are usually constipated.

In a fourth set of cases the symptoms come on more gradually, several days even elapsing before they are fully established. Such an attack is well depicted in the case reported for this issue by Dr. Parkinson.

In a fifth set of cases the disease is epidemic, several animals being attacked in the same stable, town or city. Or the epidemic may travel from stable to stable, town to town, or city to city.

These remarks are thrown out more as an incentive to veterinary surgeons to study this disease with these lesions in view, than to give a final classification; but the comparatively few cases which we have had the opportunity of closely examining have revealed the lesions already given, a knowledge of which, if our view of this disease should prove true in all cases, will suggest a plan of treatment which may restore many animals afflicted in this way to perfect health and strength.

As these examinations have led us to believe that the primary lesion is a congestion of the lumbar region of the cord, its membranes, and the spinal centres, with possibly œdema, which may be followed by myelitis, all spinal stimulants should be strictly avoided. Motor and spinal depressants, and specially those which tend to diminish the spinal irritation and congestion, should be resorted to. Physostigma Venosum, ergot, chloral-hydrate, bromide-salts, and the like. The free and early use of ergot and chloral-hydrate will probably be followed by the best results, the ergot being the best of the two.

At the same time, the kidney lesion should not be neglected, but occupy our close attention, and be treated by free purgation, diaphoretics, and non-irritating diuretics, as large draughts of water and digitalis. For it appears to the writers that many of these cases eventually die from suppression of urine, uræmic convulsions and coma.

Again, special attention should be paid to the bladder, for in a number of these cases we have found a tendency to cystitis, ulceration of the mucous membrane, and the formation of calculi. The bladder, therefore, should be thoroughly washed out at least twice a day with tepid water, containing some bland disinfecting solution, or the following can be used:

℞—Boracis, ℥ i.
Glycerin, ℥ ii.
Aquæ, ℥ iv.
M

*Sig.*—Add one ounce of the above mixture to one quart of tepid water, and thoroughly cleanse the bladder, after the urine has been drawn with a catheter. Hot fomentations over the loins may be of service, if the quantity of urine is small, but, better still, dry cupping. Cupps are hard to apply to horses; but they can be applied, and quite effectually, too, provided the hair has been previously cut close to the skin. Diaphoretics may be resorted to. As soon as the animal shows any signs of improvement, slight exercise on level ground should be instituted.

*By* WILLIAM HENRY PORTER, M.D., D.V.S.,
*Professor of Surgery at the Columbia Veterinary College and School of Comparative Medicine,*
*And* GEORGE H. PARKINSON, D.V.S.,
*Middletown, Connecticut.*

